# Follicular phase length has no influence on frozen-thawed embryo transfers in natural cycles

**DOI:** 10.1186/s13048-020-00690-z

**Published:** 2020-07-31

**Authors:** Ying Ying, Fuman Qiu, Qing Huang, Sichen Li, Haiying Liu, Jianqiao Liu

**Affiliations:** 1 Reproductive Medicine Center, The Third Affiliated Hospital of Guangzhou Medical University, 63 Duobao Road, Liwan District, Guangzhou, China; 2 The State Key Lab of Respiratory Disease, Collaborative Innovation Center for Environmental Toxicity, The School of Public Health, Guangzhou Medical University, Guangzhou, China

**Keywords:** Follicular phase length, Frozen–thawed embryo transfer, Natural cycle

## Abstract

**Background:**

Whether menstrual variability in women with regular and ovulatory cycle could affect embryo implantation remains controversial, with conflicting evidences presented in the literature. Thus, in this study, we evaluated the impact of prolonged follicular phase length (FPL) on the clinical pregnancy rate (CPR) after frozen–thawed embryo transfer (FET) in true natural cycles (NC).

**Methods:**

This retrospective cohort study utilized data from a large university-affiliated reproductive medicine center. Women undergoing true NC-FET were grouped as per their FPL type: Prolonged FPL (*n* = 127) and Normal FPL (*n* = 737). The primary study outcome was CPR in these 2 groups.

**Results:**

The FPL in the current cycle was significantly longer in the Prolonged FPL group (23.0 ± 2.4) than in the Normal FPL group (16.0 ± 2.2; *p* < 0.001). The crude CPR was significantly higher in the Prolonged FPL group (61.4%) than in the Normal FPL group (51.7%; *p* = 0.043). After adjusting for the results of potential confounders including the age, BMI, percent of optimal embryos transferred, and endometrial thickness, the difference in the CRP between the 2 groups disappeared (OR 1.28, 95% CI: 0.86–1.91, *p* = 0.232). No statistically significant difference was noted in the rates of implantation and miscarriage.

**Conclusions:**

The current FET should not be cancelled if the ovulation time exceeds the predicted period based on the length of the previous menstrual cycle in the light of no negative effect on the pregnancy outcome.

## Background

Transferring fewer embryos, minimizing the risk of ovarian hyperstimulation syndrome (OHSS) during fresh in vitro fertilization (IVF) cycles, and the potential deleterious effect of controlled ovarian stimulation on endometrial receptivity has been reported to increase the overall numbers of frozen-thawed embryo transfer (FET) cycles [1]. Although FET is a routine practice now, significant diversity exists in its practice among clinics, such as with regards to the protocols for endometrial preparation. In ovulatory patients, the natural cycle-FET (NC-FET) is preferred over programmed cycle-FET as the former involves minimization of exogenous hormone replacement [2], making it attractive to both the patients and clinicians.

The embryo quality, ploidy status, endometrial thickness, and transfer efficiency are essential for a successful FET program [3–6]. In addition, the optimal timing for NC-FET should be considered carefully with due consideration to the luteinizing hormone (LH) surge by serum monitoring in combination with a decrease in the serum estradiol level and an increase in the serum progesterone level, as well as the ultrasound detection of ovulation. Although most comparative studies have failed to reach a consensus on the definition of this event for NC-FET [7–9], it seems that different reproductive centers achieve their “ideal success rate” based on their own standards designed as per the encountered conditions. An intensive monitoring of spontaneous ovulation is essential for timely determination of NC-FET. During the monitoring period, delay in the ovulation of a considerable number of patients with regular menstrual cycle was detected, which raises the question of whether the prolonged follicular phase had a detrimental effect on the endometrial receptivity, which in turn led to implantation failure in ovulation-delayed patients.

Currently, no data are available supporting the potential role of follicular phase length (FPL) on successful FET. Thus, in the present study, we attempted to explore the relationship between delayed ovulation (defined as FPL of ≥21 days in this study, because the menstrual cycle of ≥35 day is a sign of oligomenorrhea) and the clinical pregnancy rate (CPR) in NC-FET with respect to the timing of embryo transfer based on the confirmatory detection of ovulation by ultrasonography, accompanied with serum LH surge and the expected physiological trends in the periovulatory estradiol and progesterone levels.

## Methods

### Inclusion criteria

All FETs performed at our center between January and July 2019 were reviewed for their potential inclusion. Only those patients who underwent true NC-FET, which is defined as the transfer of a frozen–thawed embryo during a spontaneous ovulatory menstrual cycle, were included in this analysis; the timing of the embryo transfer was determined based on the endogenous LH surge and the ultrasound detection of ovulation. Thus, NC-FET modified by the administration of human chorionic gonadotrophin (HCG) to trigger ovulation was excluded from the review. The exclusion criteria included patients aged > 40 years, preimplantation genetic testing (PGT) cycles, women with > 3 previously failed embryo transfer cycles, and patients with the peak endometrial thickness of < 6 mm. The study protocol was approved by the Institutional Review Board of The Third Affiliated Hospital of Guangzhou Medical University.

### NC-FET protocol

The procedures of controlled ovarian stimulation, trigger injection, oocyte retrieval, embryo culture, embryo transfer, and cryopreservation were conducted according to the standard protocols. Fertilization was achieved via conventional in vitro fertilization (IVF) or intracytoplasmic sperm injection (ICSI) based on the semen parameters and the history of prior IVF outcomes. Fresh embryo transfers were conducted either on day 3 or day 5 based on the embryo quality, number, and clinical indications. Similarly, embryos were vitrified at the cleavage or blastocyst stage, depending on the embryo quality, number, and clinical indications.

FET was performed through a natural cycle or through a programmed cycle as per the doctor’s experience and patient’s specific situation and requirements. Only those women who were undergoing true NC-FET were included in this study. During true NC-FET, transvaginal ultrasonography was performed to assess follicle development and endometrial thickness and pattern as well as for the serum monitoring of the levels of LH, estradiol, and progesterone, which began from the cycle days of 10–12 based on the length of the patient’s usual menstrual cycle, which is 2–4 days prior to the suspected ovulation date. Ultrasounds were repeated until the dominant follicle attained a diameter of ≥17 mm and a trilaminar lining of > 7 mm; meanwhile, an intensive monitoring of the serum hormone levels was performed. On detecting elevated levels of LH, ultrasonography and serum hormone testing were repeated every 24 h until the detection of ovulation of the dominant follicle. Cleavage-stage embryo transfer was conducted on day 3 and blastocyst transfer on day 5 following the determined ovulation date (considering the determined ovulation day as day 0 and transfer day as days 3 and 5 for cleavage-stage embryos and blastocysts, respectively). In addition, each patient underwent either cleavage-stage transfer or blastocyst transfer with a single, one-stage embryo.

Patients were categorized into 2 groups depending on the FPL in the present NC-FET to be analyzed: (1) Prolonged FPL group: this group included women whose FPL was ≥21 days and (2) Normal FPL group: this group included patients with FPL < 21 days.

### Embryo grading

The quality of each embryo was assessed using the following grading system: Day 3 embryos were evaluated based on the number and size of their blastomeres and the degree of fragmentation, with good quality day-3 embryos defined as 7–9 even, equally sized blastomeres with < 20% fragmentation of blastomeres. Blastocysts were graded according to the following 3 morphological parameters: inner cell mass (ICM), trophectoderm, and the degree of expansion with a hatching stage [10]. At our center, blastocysts of grade 4BB or better were defined as high-quality blastocysts. Vitrification was performed on days 3, 5, or 6 based on the development of each embryo.

### Main outcome measures and statistical analysis

The basic demographic characteristics were compared among the patients with both timely and delayed ovulations in the NC-FET groups using the Mann–Whitney U-test for continuous variables or Chi-square χ2 test for categorical variables. CPR, defined as the visualization of intrauterine gestation identified by a transvaginal ultrasound at 7 weeks of gestational age, was the main outcome of our study. The secondary outcomes included implantation rate (IR) and miscarriage. IR was defined as the proportion of transferred embryos that resulted in clinical pregnancy (the number of intrauterine gestational sac identified by transvaginal ultrasound based on the number of embryos transferred). The miscarriage rate was defined as the proportion of clinical pregnancies resulting in first-trimester pregnancy loss. Odds ratios (OR) with 95% confidence intervals (CI) were calculated and adjusted for patient’s age, body mass index (BMI), embryo grading, peak endometrial thickness, and ovulation day using the multivariate logistic regression. All tests were two-sided and performed using the SAS software (version 9.3; SAS Institute). *P* < 0.05 was considered to be statistically significant.

## Results

**Fig. 1:**
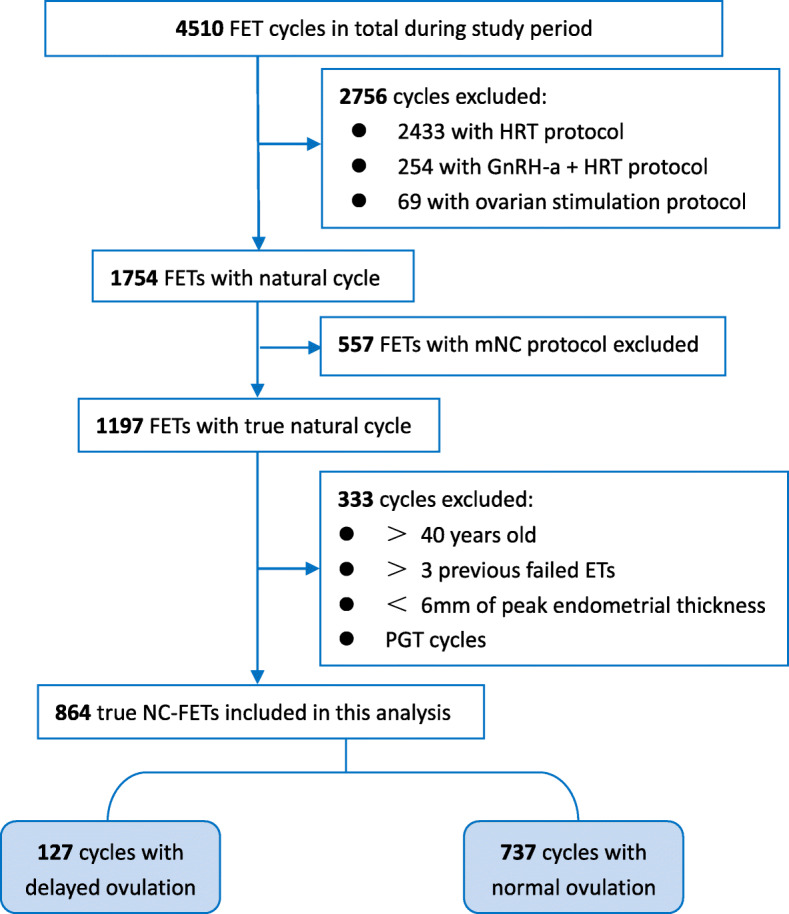
Flowchart of eligibility criteria

During the study period, a total of 4510 FET cycles were performed at our center. Among these, 864 cycles of true NC-FET were included in the present analysis, which were divided into 2 groups depending on the ovulation day of present menstrual cycle for FET (Fig. 1).

The demographic characteristics of all patients are summarized in Table 1. No significant differences were noted between the 2 groups for the patients’ age, BMI, infertility type and duration, infertility factor, gravidity, parity, number of previous failed embryo transfer cycles, fertilization method, and menstrual cycle length.

**Table 1:** Baseline characteristics of the study population

Parameters	Prolonged FPL group (*n* = 127)	Normal FPL group (*n* = 737)	*p* value
Age (years)	32.1 ± 4.22 (22–40)	32.7 ± 4.03 (22–40)	0.087
BMI (kg/m^2^)	21.8 ± 3.23 (15.3–31.9)	21.7 ± 2.84 (15.5–31.5)	0.774
Infertility type			0.659
Primary	59 (46.5%)	358 (48.6%)	
Secondary	68 (53.5%)	379 (51.4%)	
Infertility duration (years)	4.9 ± 3.1 (1–17)	4.7 ± 3.2 (1–18)	0.445
Infertility factor			0.223
Tubal factor	70 (55.1%)	366 (49.7%)	
Male factor	14 (11.0%)	126 (17.1%)	
Mixed male and female factor	31 (24.4%)	155 (21.0%)	
Unexplained	12 (9.5%)	90 (12.2%)	
Gravidity	0.98 ± 1.04 (0–4)	0.95 ± 1.09 (0–6)	0.636
Parity	0.34 ± 0.51 (0–2)	0.29 ± 0.49 (0–2)	0.314
No. of previous failed cycles	0.91 ± 0.82 (0–3)	0.97 ± 0.87 (0–3)	0.552
Fertilization method			0.696
IVF	113 (89.0%)	636 (86.3%)	
ICSI	12 (9.4%)	84 (11.4%)	
IVF + ICSI	2 (1.6%)	17 (2.3%)	
Menstrual cycle length	29.0 ± 2.85 (23–38)	28.7 ± 2.71 (21–36)	0.250

*FPL* follicular phase length

The characteristics of FET in true NC are shown in Table 2. The FPL in the current cycle was significantly longer than that in the Prolonged FPL group (23.0 ± 2.4) when compared with that in the Normal FPL group (16.0 ± 2.2, p < 0.001). No statistically significant differences were noted at the embryonal stage during transfer as well as in the number of embryos transferred between these 2 groups. Patients in the Prolonged FPL group showed a higher percent of optimal embryos transferred at the cleavage-stage (65.8% vs 53.1%, *p* = 0.044) and significantly thicker endometrial thickness (8.64 ± 1.34 vs 8.32 ± 1.37, *p* = 0.004) when compared with those in the Normal FPL group. The crude CPR was significantly higher in the Prolonged FPL group (61.4%) as compared to that in the Normal FPL group (51.7%, *p* = 0.043); however, there were no marked differences in the rates of implantation and miscarriage.

**Table 2:** Characteristics of the true NC-FET

Parameters	Prolonged FPL group (*n* = 127)	Normal FPL group (*n* = 737)	*p* value
Ovulation day	23.0 ± 2.4 (21–33)	16.0 ± 2.2 (9–20)	**<0.001**
Embryonal stage at transfer			0.591
Cleavage-stage	41 (32.3%)	256 (34.7%)	
Blastocyst	86 (67.7%)	481 (65.3%)	
No. of embryos transferred	1.52 ± 0.50 (1–2)	1.50 ± 0.50 (1–2)	0.651
Cleavage-stage	1.78 ± 0.42 (1–2)	1.66 ± 0.48 (1–2)	0.116
Blastocyst	1.40 ± .049 (1–2)	1.41 ± 0.49 (1–2)	0.750
Optimal embryos transferred			
Cleavage-stage	65.8% (48/73)	53.1% (225/424)	**0.044**
Blastocyst	76.7% (92/120)	69.3% (471/680)	0.102
Endometrial thickness	8.64 ± 1.34 (6.1–12.0)	8.32 ± 1.37 (6.0–13.8)	**0.004**
Biochemical pregnancy			0.086
Yes	80 (63.0%)	404 (54.8%)	
No	47 (37.0%)	333 (45.2%)	
Clinical pregnancy			**0.043**
Yes	78 (61.4%)	381 (51.7%)	
No	49 (38.6%)	356 (48.3%)	
Implantation rate	48.2% (93/193)	41.9% (463/1104)	0.106
Miscarriage rate	7.7% (6/78)	13.1% (50/381)	0.182

In the multivariate logistic regression model (Table 3), only the women’s age (OR 0.95, 95% CI: 0.91–0.98, *p* = 0.002) and morphologically optimal embryos transferred (OR 3.21, 95% CI: 2.31–4.47, *p* < 0.001) were considered as important independent prognostic factors for confirming clinical pregnancy. After adjusting the results for the above-mentioned potential confounders as well as the BMI and endometrial thickness, the difference in CPR between the 2 groups disappeared (OR 1.28, 95% CI: 0.86–1.91, *p* = 0.232).

**Table 3:** Multivariate logistic regression analysis of factors related to clinical pregnancy

Variables	β	SE(β)	Wald χ^2^	*P value*	OR(95%CI)
Age	−0.056	0.018	9.60	0.002	0.95 (0.91–0.98)
BMI	0.043	0.025	2.97	0.085	1.04 (0.99–1.10)
Endometrial thickness	0.057	0.054	1.16	0.281	1.06 (0.96–1.17)
Optimal embryos transferred	1.168	0.169	47.96	< 0.001	3.21 (2.31–4.47)
Ovulation time	0.244	0.205	1.42	0.232	1.28 (0.86–1.91)

## Discussion

Our data showed that women with a follicular phase length ≥ 21 days in the current FET cycle shared similar CPRs with those who ovulated within a normal time range (with a follicular phase length < 21 days in the current FET cycle), and the only important independent prognostic factors for clinical pregnancy were found to be the women’s age at the time of FET (all embryos were warmed and transferred within a year since vitrification) and the transfer of morphologically optimal embryos, this finding is supported by those of a few previous studies [11, 12].

As we know, one of the commonly used approaches for FET is the NC monitoring, wherein the endometrium undergoes morphological and biochemical changes mainly mediated by exposure to endogenous estrogen and progesterone, and such changes are essential for implantation. The hormone changes occurring around the ovulation period have been an area of interest for decades [13]. In order to achieve an optimal outcome in NC-FET, the transfer of the thawed embryo(s) should be performed at the time of the highest endometrial receptivity.

Reportedly, even in women with regular and ovulatory cycles, the spontaneous pregnancy rates are affected by the menstrual cycle characteristics, such as its length, variability, and the luteal phase length [14–16]. It has been shown that 84% of cycle length variability is due to variation in follicular phase length [17], short follicular phases are associated with higher estrogen in follicular phase [18]. The appearance of shortened follicular phase lengths is accompanied by changes in gonadotropin and ovarian hormones among aging women, including increased follicular phase estrogen [19, 20]. During this same time, women experience decreased fertility. Therefore, short menstrual cycles are associated with decreased fecundity [15].

However, there is little information on the association of prolonged follicular phases with fertility. One study reported in 1991 showed that implantation rates did not vary over a wide range of follicular phase lengths following transfers of embryos created with fresh donor eggs [21], however in this study, the endometrium preparation was performed by using hormone replacement therapy. A recent study indicated the lack of association between the menstrual cycle characteristics (such as the length, variability, and predicted luteal phase length) and the rate of live birth after FET in the natural cycles with progesterone supplementation [22], however, one obvious shortcoming of this study is that the grouping is based on calculating the predicted luteal phase length of previous menstrual cycle, not the current FET cycle.

Thus, further research is warranted to investigate the influence of menstrual cycle characteristics on embryo implantation. With the development of FET technology, our focus has shifted to the details of the treatment process for the optimization of the success rate of FET. The present study was conducted to investigate the potential impact of prolonged FPL of true NC-FET on the outcome of clinical pregnancy in women with regular menstrual cycle, and our data indicated no influence of prolonged follicular phase length on the rates of clinical pregnancy, implantation and miscarriage in patients undergoing true-NC-FET.

To the best of our knowledge, this is the first study to address the potential influence of FPL on the CPR of NC-FET. However, the present study is limited by its retrospective nature, with the potential for selection bias, heterogeneity introduced by the implementation of natural cycle protocol by different performers, confounders such as the differences in the percent of optimal embryos transferred and the peak endometrial thickness between the 2 groups, as well as the lack of data on the later stage of pregnancy outcomes (such as the ongoing pregnancy rate and live birth rate). Fortunately, because of the large number of FET cycles in our center (more than ten thousand FET cycles per year on average were completed in our center), although it is a retrospective study, the inclusion criteria of this study are very strict, which might have reduced the confounding factors associated with many such variables that could influence the outcomes.

There is a need for further well-designed prospective studies of a larger sample size to verify the influence of FPL on the success of embryo implantation, and to provide more information of clinical significance, including ongoing pregnancy rate, live birth rate, and the incidence of delayed ovulation in women with regular menstrual cycles.

## Conclusions

Delayed ovulation does not adversely affect CPR after FET in NC, indicating that the current FET should not be cancelled if the ovulation time exceeds the predicted period based on a previous menstrual cycle length in light of no negative effect on the pregnancy outcome.

## Data Availability

The datasets used and/or analysed during the current study are available from the corresponding author on reasonable request.
